# The Impact of Meal Timing on Risk of Weight Gain and Development of Obesity: a Review of the Current Evidence and Opportunities for Dietary Intervention

**DOI:** 10.1007/s11892-022-01457-0

**Published:** 2022-04-11

**Authors:** Rochelle Davis, Michelle Rogers, Alison M. Coates, Gloria K. W. Leung, Maxine P. Bonham

**Affiliations:** 1 Department of Nutrition, Dietetics and Food, Monash University, Level 1, 264 Ferntree Gully Road, Notting Hill, Melbourne, VIC 3168 Australia; 2 Behaviour-Brain-Body Research Centre, UniSA Justice & Society, University of South Australia, Adelaide, South Australia Australia; 3 Alliance for Research in Exercise, Nutrition and Activity Research Concentration, UniSA Allied Health & Human Performance, University of South Australia, Adelaide, South Australia Australia

**Keywords:** Meal timing, Obesity, Time-restricted eating, Energy expenditure

## Abstract

**Purpose of Review:**

The aim of this short review is to provide an updated commentary on the current literature examining the impact of meal timing on obesity and weight gain in adults. The potential mechanisms, including novel and emerging factors, behind timing of food intake across the 24-h period in the development of obesity, and dietary strategies manipulating meal timing to ameliorate weight gain are also explored.

**Recent Findings:**

Dietary patterns that feature meal timing outside of the regular daytime hours can contribute to circadian disruption as food is metabolised in opposition to internal daily rhythms and can feedback on the timekeeping mechanisms setting these rhythms. Epidemiological evidence examining the impact of late meal timing patterns is beginning to suggest that eating at night increases the risk of weight gain over time. Mechanisms contributing to this include changes to the efficiency of metabolism across the day, and dysregulation of appetite hormone and gut microbiota by mis-timed meals.

**Summary:**

When meals are eaten, in relation to the time of day, is increasingly considered of importance when implementing dietary change in order to address the growing burden of obesity, although further research is required in order to determine optimal patterns.

## Introduction

Rates of chronic disease are increasing globally, and represent a significant societal and economic impact due to the curtailment of disease-free years of life [1]. Increased adiposity is a key mediating factor for development of chronic cardio-metabolic diseases such as type 2 diabetes and cardiovascular disease and has experienced a similar upward trajectory, with now approximately 39% of adults worldwide being overweight and 13% having obesity [2]. Further to the other well-known aspects of dietary patterns that impact on weight maintenance, such as the type and amount of foods eaten, the temporal patterns of food consumption is a factor increasingly being considered of importance in the efforts to further understand the aetiology and prevention of obesity. This is in part due to the increased propensity for weight gain observed in those with mis-timed eating patterns, i.e. eating occasions extending in to the night hours, when the body is usually primed for rest [3]. Night shift workers are a case in point, being frequently active and eating at night in the course of their employment, and experiencing higher rates of cardio-metabolic disease [4]. The human body is highly attuned to the cycling of day and night (circadian rhythm), with daily fluctuations in many physiological processes, including insulin sensitivity, occurring in anticipation of this routine environmental change [5]. Mis-timed food intake in relation to the day/night cycle can therefore cause circadian disruption, which has been hypothesised to contribute to the development of obesity and associated cardiometabolic disorders [6].

Several mechanisms as to how meal timing contributes to circadian disruption leading to an increased risk of overweight/obesity and as a result, poorer metabolic health, have been proposed. Firstly, food consumption that is out of synchronisation with typical light/dark cycles could lead to impaired satiety hormone production (i.e. leptin and ghrelin) resulting in increased food consumption [7]. Additionally, the timing of food intake may affect energy expenditure, with diet induced thermogenesis, i.e. the increase in metabolic rate following the consumption of food, decreasing throughout the day [8–10]. The cycling of feed/fast and sleep/wake behaviours in relation to light/dark cycles impacting gut microbiota is also considered as an emerging mediating factor contributing to increased adiposity [11]. Lastly, we also examine the potential for manipulating the timing of nutrient intake and fasting periods as a therapeutic intervention to ameliorate weight gain and improve metabolic health.

The aim of this review is to assess the current literature examining the evidence surrounding impact of meal timing (as defined for this review by the time day or night at which food is consumed) on obesity and weight gain in adults. The potential mechanisms behind timing of food intake in the development of obesity, and dietary strategies manipulating meal timing to ameliorate weight gain are also explored.

## Epidemiology of Obesity and Weight Gain in those who Habitually Eat at Night or Later in the Day

As a population, there has been a shift in the timing of when we eat with observational data reporting consumption of a greater proportion of energy towards the latter half of the day [12]. Data from the 2011–2015 NHANES survey indicated that up to 45% of daily energy intake comprises evening meals and snacks [13]. These changes to our lifestyle may have implications for weight maintenance. A number of observational studies have explored the association between meal timing and weight status drawing associations between long-term weight gain and the time at which the majority of energy is consumed even when total energy intake is adjusted for. The evidence is more compelling when we examine findings from populations who have little choice but to eat at night such as shift-workers.

In a cohort of 1245 individuals, participants who consumed ≥ 48% of their daily energy intake at dinner were twice as likely to be obese at 6-year follow-up, even after adjustment for variations in energy intake, physical activity and BMI at baseline. (OR = 2.33; 95% CI: 1.17; 4.65) [14]. In a separate study of 239 participants, Wang et al. determined that those who consumed ≥ 33% of total energy in the evening (compared to < 33%) were twice as likely to be overweight or obese (OR = 2.00; 95% CI: 1.03; 3.89). with these findings being adjusted for variations in total energy intake and physical activity [15]. In a review, Fong et al. [16] summarised nine cross-sectional and one prospective cohort studies, and found that four of the ten studies observed a positive association between increased evening intake and increased body weight. Of the remaining studies, five found no association and one a negative association between increased evening food consumption and increased body weight. Important to note, is the high degree of heterogeneity noted by the authors observed in both the assessment of exposure and outcome variables which may have contributed to the inconsistency in findings. Conversely, consuming a greater proportion of energy at lunch, i.e. earlier during the day, appeared to reduce the risk of weight gain at 3.5-year follow-up (OR = 0.62, 95% CI: 0.47; 0.80) [17].

Eating late into the day and the overnight period is common for 15–20% of the population employed in shift work roles, as such data from this unique population group can assist our understanding of associations between meal timing and body weight. A number of retrospective studies have reported a link between exposure to shift work and weight gain [18–20] and a recent meta-analysis found a significantly increased prevalence of overweight/obesity in shift workers from the 22 cross-sectional studies included as part of the review [21]. Increases in weight have been observed in the absence of increased energy intake [22] implicating meal timing as a possible mechanism for the change in weight.

Inadequate sleep duration is another factor potentially mediating the increase in weight, as night shift patterns have been shown to be unfavourable to sleep [23]. Shorter sleep duration is known to contribute to development of obesity and type 2 diabetes [24], although has been underexplored in this population and presents a limitation in assessing the relationship between shift work and obesity risk. This would be important to examine in the future as night-time eating is more prevalent in short sleepers and can both be a consequence of, and effectuate, short sleep duration [25].

While there are clear limitations in methodological difference in dietary data collection and synthesis of findings due to heterogeneity in the classification of night eating exposure and outcomes measured, this evidence is starting to suggest that the time of day when meals are consumed can impact body weight and that night eating may lead to weight gain. Future prospective cohort studies which comprehensively characterise the timing of meals, in addition to more traditional markers of diet quality, are needed before strong conclusions can be formed in regards to the contribution of meal timing patterns to obesity.

The mechanisms behind the observed increase in risk of obesity and weight gain in populations frequently eating at night are likely to be multifaceted, and, as suggested in the epidemiological evidence, not explained by disturbances to energy intake alone. It is therefore important to consider the impact of the temporal distribution of meals, in relation to the daily rhythms in physiological processes generated by the body’s internal clocks, on homeostatic mechanisms that control weight maintenance.

## Metabolic Processes are Governed by the Circadian Clock and Mis-timed Meals can cause Circadian Disruption

Humans are diurnal animals, with activity periods typically occurring during the daytime hours. Correct alignment of active versus rest periods with the constant cycling of light and dark from the Earth’s rotation is achieved through an adaptable endogenous timekeeping mechanism, the master clock. This resides in the suprachiasmatic nucleus and is entrained by the perception of light in the retina [26]. In turn, this master clock synchronises molecular peripheral clocks, oscillations in negative and positive arms of core clock gene transcripts found in every cell [27]. These oscillations of the peripheral clock regulate output genes which coordinate many physiological processes, including hormone secretion and metabolism, thus ensuring they are timed to occur in the correct phase of activity or rest [28]. Approximately 15% of the metabolome oscillates across the day [29]. The regulation of metabolic responses has also shown to be different across the day, with eating at night eliciting a different post-meal regulatory response in gene expression compared to eating during the day, with less genes involved in lipid metabolic pathways activated at night [30]. Notably, homeostasis of glucose and lipid blood concentrations are under circadian regulation, with decreased glucose [31] and lipid clearance [32] during the night-time hours.

**Fig. 1:**
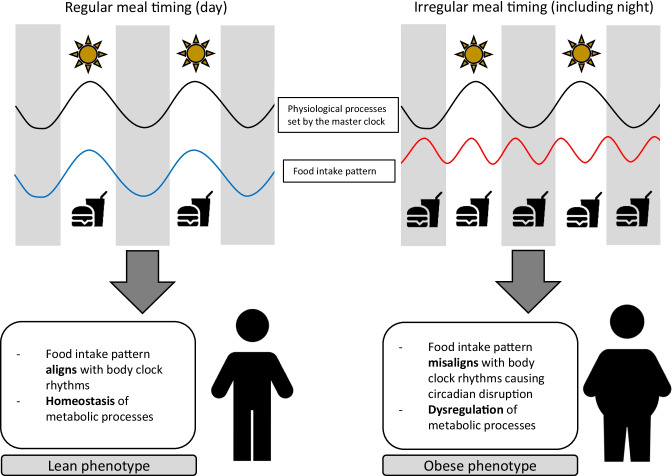
Meal timing impacting the development of obesity. Select clipart created by Peter van Driel from the Noun Project

Mistimed eating in relation to body clocks causes circadian disruption. This disruption can occur on both an external level i.e. a misalignment of feed/fast cycles with the body clocks, and an internal level, by food acting as an entraining cue for peripheral clocks, causing an uncoupling of the peripheral clocks in metabolic tissue with the master clock rhythm set to daylight [33]. Animal models of circadian disruption i.e. active and eating at night, in inversion to the internal rhythms set by the master clock, have produced increases in body weight after meals were timed in the usual rest phase compared to the active phase, even in isoenergetic conditions [34]. Although experimental evidence in humans is lacking, circadian disruption has been linked to metabolic disturbances; 8 days of a circadian disruption protocol in ten adults caused a 22% increase in insulin levels (*p* = 0.006) and 6% increase in glucose (p < 0.001) [35]. Additionally, in dietary weight loss interventions that compared differences in the temporal patterns of meals found lower success with achieving weight loss in those people who had late meal timings [36]. The internal body clock broadly impacts many aspects of metabolic regulation which are involved in the development of obesity (Fig. 1), it is important therefore to further explore the potential mechanisms which may facilitate this.

## Energy Expenditure Changes Across the 24-h Day with Decreased Energy Expenditure at Night

Integral to the development of obesity is the imbalance of energy intake relative to energy expenditure. Total energy expenditure (TEE) is comprised mainly of the resting metabolic rate (RMR); the energy used in the maintenance of homeostasis, and to a lesser extent the thermic effect of food (TEF); energy used for the digestion, absorption and catabolism necessary after a meal. There is evidence that both of these components fluctuate over the course of the day, which, given that RMR and TEF combined make up approximately 80% of TEE, could help explain the difficulties in achieving weight maintenance when meal timing is misaligned with these fluctuations. In a well-controlled laboratory protocol which assessed circadian rhythms in RMR, measured by indirect calorimetry after fasting, it was found that RMR was lowest late at night (0500 h) and highest around 12 h later, in the late afternoon [37••]. A similar peak and nadir during the day and night respectively was observed in TEF, with a study examining the increase in energy expenditure after eating a meal at 8 pm compared to 8 am, finding post-meal energy expenditure increases were 380 kJ (95% CI: 170; 590 kJ, p < 0.001) lower at night, despite identical fasting, rest, and physical activity conditions [10]. A recent review which synthesised evidence from experimental studies measuring TEE changes across the 24-h day found overall there was some evidence to suggest the thermic effect of food was less efficient over the night hours, however further studies were needed to elucidate the magnitude of potential differences in resting metabolic rate [38]. Although the thermic effect of food contributes a relatively small component to total energy expenditure, skewing energy intake towards later in the evening, when TEF is least efficient, may contribute to cumulative weight gain if continued in the long term.

## Variation Across the Day in Hunger and Satiety Hormones may Impact Weight Regulation

Appetite regulating hormones ghrelin and leptin exhibit daily rhythms of synthesis and secretion [39]. Leptin, secreted from white adipocytes, is implicated in the regulation of hunger, food intake and energy balance, while ghrelin stimulates appetite, fat production, and body growth—leading to increased food intake and body weight. The circulating level of ghrelin is mainly regulated by nutritional status, rising by fasting and decreasing after food ingestion [40]. The circadian peak in ratings of hunger occurs in the biological evening, and the circadian trough in hunger ratings occurs in the biological morning [41] corresponding with ghrelin levels being elevated at night.

Obesity is associated with impairments in circadian rhythms of appetite regulating hormones including leptin where the nocturnal peak (close to midnight) is delayed by ~ 3 h compared with normal-weight, sex-matched control participants [42]. Weight loss can restore this deregulation [43]. Participants in a 12-week hypocaloric intervention who achieved a mean weight loss of 10%, had partially restored daily rhythms of leptin and a modified ghrelin rhythm, but appetite sensations were barely modified which is indicative of leptin resistance. Despite some improvements in hormonal regulation associated with weight loss, when excessive adipose tissue remains, these hormones still do not display normal physiological functionality [44].

Meal timing can influence hormonal appetite/hunger responses [45] and disruption of normal rhythms can contribute to elevated adiposity [46]. Evidence from a tightly controlled laboratory study that manipulated the timing of meals, but not the duration of the eating window, in conjunction with sleep timing, found higher glucagon-like peptide concentrations in response to a meal with early sleep and mealtimes, suggesting improved satiety compared with the late meal condition [47]. In contrast, earlier sleep and mealtimes were associated with higher ghrelin concentrations and did not influence leptin concentrations. The combination of normal sleep and mealtimes had a reducing effect on food intakes.

The influence of meal timing on energy regulation has been demonstrated in a recent study of obese participants undergoing weight loss. Participants were classified as either late or early eaters and the impact of timing of habitual meals on weight loss effectiveness was compared. Late eaters had higher concentrations of leptin in the morning compared with early eaters, but no difference between groups in ghrelin. Consistent with measured satiety hormones, compared with early eaters, late eaters reported being less likely to have a high appetite in the morning. The late eaters had less success with weight loss that equated to a difference of 1.5 kg over a 19-week intervention [48••].

## Contribution of Diurnal Changes in the Gut Microbiome to Weight Gain and Obesity

The gut microbiota (e.g. the community of different microorganisms residing in the gastrointestinal tract), and the microbiome (e.g. these microorganisms and their genes), influence the health of their human hosts including the development of obesity, and its further complications [11]. Metabolites produced by the gut microbiota act as a link between gut microbiota and its host and the composition of the gut microbiota is dynamic and undergoes daily cyclic fluctuations [49]; with this rhythm being affected by light/dark cycles, sleep/wake cycles, dietary composition and eating patterns.

There are complex relationships between circadian rhythms, gut microbes, and energy metabolism. Several changes in gut function are observed with circadian disruption including enhanced gut permeability, resulting in the dysfunction of gut barrier and alteration of gut microbiome [50, 51]. Changes to both the abundance and diversity of the microbiota alter dietary metabolism and the extent to which humans are able to derive energy from foods [52]. Studies in mice indicate peaks in the abundance of the bacterial phylum, *Firmicutes*, immediately after meals, with the more beneficial phyla *Bacteroidetes* and *Proteobacteria* only able to bloom during periods of fasting [51]. The balance between these phyla also changes in humans; a 20% increase in *Firmicutes* with a corresponding decrease in *Bacteroides* was associated with an increased energy harvest from the host diet of 630 kJ per day, potentially resulting in a body weight gain of 5 kg over 1 year [52]. Furthermore, obese populations (both human and rodent) have been found to have a shift in their gut microbiome compared with lean population towards an imbalance such that the *Bacteroidetes* phylum is lower and the *Firmicutes* phylum is higher which would in turn, lead to greater energy being obtained from the host diet of obese people [53].

If circadian rhythms are constantly disrupted, as occurs with frequent late or night-time eating, this has been shown to uncouple peripheral circadian clocks in intestinal epithelial cells [54] which can then reduce fuel utilisation and lead to fuel storage in adipose tissue. Thus, the alteration in fuel utilisation has been proposed as a contributor to weight gain in shift workers who routinely face circadian misalignment. Time restricted feeding in rodent models partially restores this misalignment and in turn has been shown to influence the improve host metabolic efficiency [55]. Thus, time restricted eating (TRE) has potential as a strategy to help not only with weight management through reduced energy intake but also through regulation of the circadian rhythms of microbiota.

## Dietary Strategies that Manipulate Meal Timing to Avoid Food Intake Overnight to Promote Weight Loss

Evidence summarised above indicates that the timing of when meals are eaten in relation to the day/night cycle can negatively impact long-term energy balance, but on the flip-side also presents a novel opportunity for intervention, as meal timing can be readily manipulated through behaviour modification to explore the potential effect on weight loss. In the last decade an increasing body of research has explored TRE, which restricts energy intake to a specified period of the 24-h day, as a promising diet strategy to improve metabolic risk markers. TRE diets can differ in the length of feeding window and the clock hours at which feeding occurs. Despite these variations in protocols, two recent meta-analyses have synthesised TRE studies in the literature, to elucidate whether such meal timing strategy has an effect on body weight changes in humans.

Meta-analysis by Moon et al. [56] collated studies that compared TRE (feeding window: 4 to 12 h) to regular diets, i.e. those with unrestricted meal times, in healthy individuals and individuals with metabolic disorders. Only intervention studies were included, but no limits were placed on the intervention’s duration. The overall meta-analysis of 12 studies reported TRE promoted significant weight loss (mean difference: − 0.90 kg; 95% CI: − 1.71 to − 0.10 kg); however, statistical significance was lost in the sensitivity analysis that removed an impactful study [56]. The effect of TRE on weight loss seems to be more prominent in those with a higher BMI or with metabolic disorders. As observed in their subgroup analyses, TRE had no effect on body weight in healthy individuals (*n* = 7 studies; mean difference: 0.17 kg; 95% CI: − 0.81 to 1.15) but led to significant weight loss in those with metabolic disorders (*n* = 5 studies; mean difference: − 3.19 kg; 95% CI: − 4.62 to − 1.77) [56]. This finding is supported by meta-analysis by Pellegrini et al. [57], which also collated studies that compared TRE (feeding: 4 to 12 h) to diet strategies with no meal time restrictions, but included both observational and intervention studies. In their subgroup analysis of five intervention studies, four of which involving healthy individuals, Pellegrini et al. reported TRE had a statistically significant but clinically negligible effect on weight loss (weighted mean difference: − 0.38 kg; 95% CI: − 0.71 to − 0.05) [57].

Considering the findings from these two meta-analyses and the clinical indications of weight loss, it seems of importance to also examine the effect of TRE on weight loss in individuals with overweight and obesity. Only a few recent intervention studies have investigated this specifically, as the majority of older articles in this literature are observational studies of Ramadan fasting [57, 58]. The TREAT trial was a 12-week randomised controlled trial (RCT), which compared TRE (feeding 1200 h to 2000 h) to consistent meal times (3 meals/day) in 105 overweight adults [59]. Only recommendations on meal timing were provided, with no advice on energy restriction, physical activity or diet quality. Findings reported no significant differences in body weight or estimated energy intake changes between the two groups [59]. In contrast, the 12-week intervention study by Gabel et al. reported significant weight loss in obese adults (*n* = 46) following the TRE diet (feeding 1000 h to 1800 h) compared to historical controls (mean ± SEM: − 2.6% ± 0.5; p < 0.001) [60]. However, a concurrent reduction in energy intake was observed in the TRE group (− 1432 ± 223 kJ/ day; *p* = 0.04). A similar pattern was observed in the 8-week RCT by Cienfuegos et al., which compared three diet strategies in 58 adults with obesity: a 4-h TRE (1500 h–1900 h), a 6-h TRE (1300 h–1900 h) and a control group (maintain usual eating habits) [61]. Significant weight loss was observed in both TRE groups compared to controls (both − 3.2% ± 0.4%; p < 0.001), with no significant difference between the TRE groups. A greater reduction in energy intake was observed in both TRE groups compared to controls (4-h TRE: − 2209 ± 427 kJ/day, *p* = 0.02; 6-h TRE: − 2368 ± 594 kJ/day, *p* = 0.01), with no differences between TRE groups. Based on the findings from these studies, it can be speculated that TRE promotes weight loss through limiting feeding opportunities thereby inadvertently reducing energy intake, rather than leveraging the potential metabolic benefits of eating during the day-time hours.

The effect of eating earlier during the day on weight changes was specifically examined in a recently published 12-week RCT, involving 75 women with overweight or obesity [62••]. All participants followed a weight loss regime with the same magnitude of daily energy restriction and increased physical activity, but were separated into two groups: Early dinner (last meal at 1900 h to 1930 h) and late dinner (last meal at 2230 h to 2300 h). Although both groups experienced weight loss, a significantly greater weight loss was observed in the early dinner group (mean ± SD: − 6.8 ± 2.0 kg) compared to the late dinner group (mean ± SD: − 4.9 ± 2.3 kg; p < 0.001). Such potential benefit of avoiding night time food intake should be further investigated, especially in populations that typically eat during the night such as shift workers. A recent pilot crossover RCT indicated that a small overnight fasting window between 0100 and 0600 h, may be able to elicit a small reduction in body weight in overweight night shift workers [63]. The optimum window of time, both in duration and timing across the day, needed to produce a reduction in body weight is worth further consideration in order to balance beneficial effects with the practicality and sustainability of very short feeding windows.

## Conclusion

The literature summarised in this review highlights emerging evidence that the timing of food intake can impact weight gain and increased adiposity, with night and later meal timings negatively impacting weight regulation favouring the development of obesity over time. The decreased efficiency of the thermic effect of food at night, although a small contribution to overall energy balance, nevertheless may play a part in decreased energy expenditure and weight gain. In addition to this, daily fluctuations in appetite hormones and gut microbiota suggest dysregulation by disturbed meal timing. Following a dietary pattern with frequent eating at night, in the long term, may increase risk of weight gain, although currently robust conclusions in relation to the effect of meal timing patterns on obesity risk cannot be arrived at as a predominance of cross-sectional studies in the epidemiological evidence pose inherent limitations in the assessment of causality, especially so in determining impact on weight change as reverse causality cannot be ruled out. It is thus important for future cohort studies examining the relationships between dietary patterns and disease outcomes to capture accurate information on the temporal timing of meals. Investigating meal timing interventions is of particular importance considering the body clocks, responsible for regulation of metabolic processes, constant entrainment to daylight negates the potential for adaptation to a habitual nocturnal dietary pattern. There is promising evidence that controlling the time of day meals are consumed can benefit those with existing obesity to aid with weight regulation although further research is needed to investigate this as a preventative dietary strategy in healthy weight individuals. Consideration should be given in these protocols to match the energy density of diets, so that effects of night and late eating, over and above increases to energy intake, can be elucidated.
